# Antidiabetic activity of flower buds of *Michelia champaca* Linn

**DOI:** 10.4103/0253-7613.45151

**Published:** 2008

**Authors:** E. Edwin Jarald, S.B. Joshi, D.C. Jain

**Affiliations:** Department of Pharmacognosy, B. R. Nahata College of Pharmacy and Research Centre, Mandsaur, Madhya Pradesh, India

**Keywords:** Alloxan, antidiabetic activity, hypoglycemic activity, lipid profile, *Michelia champaca*

## Abstract

**Objective:**

To identify the antihyperglycemic activity of various extracts, petroleum ether (60-80°), chloroform, acetone, ethanol, aqueous and crude aqueous, of the flower buds of *Michelia champaca*, and to identify the antidiabetic activity of active antihyperglycemic extract.

**Materials and Methods:**

Plant extracts were tested for antihyperglycemic activity in glucose overloaded hyperglycemic rats. The effective antihyperglycemic extract was tested for its hypoglycemic activity at two-dose levels, 200 and 400 mg/kg respectively. To confirm its utility in the higher model, the effective extract of *M. champaca* was subjected to antidiabetic study in alloxan induced diabetic model at two dose levels, 200 and 400 mg/kg respectively. The biochemical parameters, glucose, urea, creatinine, serum cholesterol, serum triglyceride, high density lipoprotein, low density lipoprotein, hemoglobin and glycosylated hemoglobin were also assessed in the experimental animals.

**Results:**

The ethanolic extract of *M. champaca* exhibited significant antihyperglycemic activity but did not produce hypoglycemia in fasted normal rats. Apart from this extract, the crude aqueous and petroleum ether extracts were found active only at the end of the first hour. Treatment of diabetic rats with ethanolic extract of this plant restored the elevated biochemical parameters significantly (*P*<0.05) (*P*<0.01) and the activity was found dose dependent.

**Conclusion:**

This study supports the traditional claim and the ethanolic extract of this plant could be added in traditional preparations for the ailment of various diabetes-associated complications.

## Introduction

Diabetes mellitus, a chronic metabolic disorder, has now become an epidemic, with a worldwide incidence of 5% in the general population. The number of people suffering from diabetes has soared to 246 million and the disease now kills more people than AIDS.[1] Decreased physical activity, increasing obesity, stress and changes in food consumption have been implicated in this increasing prevalence in the past two decades.[2] In conventional therapy, Type I diabetes is treated with exogenous insulin and Type 2 with oral hypoglycemic agents (sulphonylureas, biguanides etc).[3] Though different types of oral hypoglycemic agents are available along with insulin for the treatment of diabetes, there is an increased demand by patients to use natural products with antidiabetic activity.[4] Since time immemorial, patients with non-insulin dependent diabetes have been treated orally in folk medicine, with a variety of plant extracts. In India, a number of plants are mentioned in ancient literature (Ayurveda) for the treatment of diabetic conditions.

Flower buds of *Michelia champaca* Linn. belonging to the family Magnoliaceae is commonly used by many traditional healers (e.g., Dr. G. Elias, Reg. No. 01323, Leela Vilasam Siddha Varma Vaidyasalai, Karungal, Tamil Nadu) in most of the herbal preparations for diabetes[5] and kidney diseases.[6] Traditionally, it is being used in fever, colic, leprosy, post partum protection[7] and in eye disorders.[8] It has been reported to possess antipyretic, anti-inflammatory,[9] insecticidal,[10] antimicrobial[7] and leishmanicidal activities.[11] The active constituents reported in this plant are alkaloids, saponins, tannins, sterols, flavonoids and triterpenoids.[7]

In Ayurveda, traditional usages of plants are generally and most commonly in the form of their aqueous extracts only. Concurrently, some of the papers searched focus testing plants in their ethanolic or aqueous extracts and some have also reported activity in petroleum ether, benzene and chloroform extracts.[12–14] Keeping these facts in view, the present study was undertaken to create a scientific base for the traditional use of the extract of *M. champaca* as an antidiabetic in diabetes associated complications, and to identify the active antidiabetic.

## Materials and Methods

### Plant material

Flower buds of *M. champaca* were collected in March 2006 from Tamil Nadu, India. The taxonomical identification of the plant was done by Dr. H. S. Chatree, Botanist, Government Arts and Science College, Mandsaur, India. The voucher specimen (BRNCP/M/003/2006) was deposited in the Herbarium of the Department of Pharmacognosy, B. R. Nahata College of Pharmacy, Mandsaur.

### Preparation of extracts

Dried and powdered plant material (500 g) was successively Soxhlet extracted with petroleum ether (60-80°), chloroform, acetone, ethanol and water for 72 h each. Crude aqueous extract of this plant was prepared separately by boiling the plant material (25 g) with 200 ml of water for 15 min. The obtained extracts were evaporated in vacuum to give residues and their percentage yields were determined.

### Phytochemical screening

In order to determine the presence of alkaloids, glycosides, flavones, tannins, terpenes, sterols, saponins, fats and sugars, a preliminary phytochemical study (color reactions) with various plant extracts was performed.[15]

### Animals and treatment

Healthy Wistar rats of either sex (150-180 g), with no prior drug treatment, were used for the present studies. The animals were fed with commercial pellet diet (Kamadenu Agencies, Bangalore, India) and water *ad libitum.* The animals were acclimatized to laboratory hygienic conditions for 10 days before starting the experiment. Animal study was performed in the Division of Pharmacology, B R Nahata College of Pharmacy, Mandsaur, after approval from the Institutional Animal Ethics Committee (registration number 918/ac/05/CPCSEA).

### Acute toxicity studies

The acute toxicity test of the extracts was determined according to the OECD (Organization for Economic Co-operation and development) guidelines No. 420. Female Wistar rats (150-180 g) were used for this study. After the sighting study, starting dose of 2000 mg/kg (p.o.) of the test samples was given to various groups containing five animals in each group. The treated animals were monitored for 14 days, for mortality and general behavior. No death was observed till the end of the study. The test samples were found to be safe up to the dose of 2000 mg/kg, and, from the results, 400 mg/kg dose was chosen as the maximum dose for further experimentation.

### Antihyperglycemic activity

Antihyperglycemic activity was studied in glucose overloaded hyperglycemic rats.[16] The animals were divided into eight groups (n = 5). Group 1 was treated with 5 mg/kg of glibenclamide and the negative control group animals; group 2, received only vehicle; the remaining six groups were treated with 400 mg/kg of various extracts of plant suspended in 1% Tween 80. Zero hour blood sugar level was determined from overnight fasted animals. After 30 min of the drug treatment (p.o.), the animals were fed with glucose (4 g/kg) and blood glucose was determined after 0.5, 1, 2, and 3 hours of the glucose load. Blood glucose concentration was estimated by the glucose oxidase enzymatic method, using a commercial glucometer and test-strips (Accu-chek Active™ Test meter).

### Hypoglycemic activity

The animals were classified in to four groups (n = 5). Group 1 was kept as control, and was given a single dose of 0.5 ml/100 g of the vehicle; group 2 was treated with glibenclamide (5 mg/kg) as the hypoglycemic reference drug. Groups 3 and 4 were treated with ethanolic extract at two dose levels (200 and 400 mg/kg) (p.o.), as mentioned in Table 2. Blood samples were collected from the tail tip at 0 (before oral administration), 0.5, 1, 2, and 3 h after administration.[17] The blood sugar level was measured using Accu-chek Active™ Test strips in Accu-chek Active™ Test meter.

**Table 2:** Effect of ethanolic extract of *M. champaca* in fasted normal rats

*Treatments*	*Dose mg/kg*	*Blood glucose concentration (mg/dl)*				
		
		*0th h*	*1/2 h*	*1 h*	*2 h*	*3 h*
Normal control	--	84.40 ± 2.32	83.00 ± 1.80	81.80 ± 2.42	82.20 ± 1.70	80.20 ± 2.38
Glibenclamide	5	82.00 ± 2.15	45.20 ± 3.86**	36.60 ± 1.43**	33.20 ± 1.39**	35.00 ± 3.16**
MC-E	200	86.20 ± 2.44	83.00 ± 2.10	82.40 ± 2.58	84.40 ± 2.58	83.40 ± 2.66
	400	84.20 ± 3.10	82.60 ± 3.18	83.40 ± 2.55	82.40 ± 2.40	80.80 ± 3.01

Each value represents the mean ± S.E.M. of five observations.

** *P*<0.01 vs control, MC -  *Michelia champaca*, E - Ethanol

### Antidiabetic activity in alloxan induced diabetes model

Alloxan induced diabetic model was selected to confirm the effectiveness of active antihyperglycemic extract in experimental diabetic conditions. Diabetes was induced in rats by injecting 120 mg/kg of Alloxan monohydrate intraperitoneally in 0.9% w/v NaCl to overnight-fasted rats. The rats were then kept for the next 24 h on 10% glucose solution bottles, in their cages, to prevent hypoglycemia. After 72 h of injection, fasting blood glucose level was measured. The animals that did not develop more than 300 mg/dl glucose levels, were rejected.[18,19] The selected diabetic animals were divided into four groups (n = 5) and one more group of normal non-alloxanised animals was also added to the study. Group 1 was kept as normal control (non-alloxanised rats), which received a single dose of 0.5 ml/100 g of the vehicle; group 2 was kept as negative control, alloxan induced and received a single dose of 0.5 ml/100 g of the vehicle; group 3, diabetic induced, was treated with glibenclamide (5 mg/kg) as the reference drug. Groups 4 and 5, diabetic induced were treated with ethanolic extract that exhibited antihyperglycemic at two dose levels (200 and 400 mg/kg), as mentioned in Table 3. The treatment was continued for seven consecutive days (p.o.). At the end of the 7^th^ day, the rats were fasted for 16 h and blood parameters were determined.

**Table 3:** Effect of ethanolic extract of *M. champaca* on biochemical parameters

*Parameters*	*Experimental groups*				
	
	*Normal control*	*Diabetic control*	*M. champaca-ethanol*		*Glibenclamide*
				
			*5mg/kg*	*200 mg/kg*	*400 mg/kg*
Blood glucose	81.40 ± 3.22**	512.00 ± 15.29	320.00 ± 14.40**	298.20 ± 12.20**	124.40 ± 7.84**
S. urea	30.20 ± 1.77**	279.00 ± 14.01	165.30 ± 6.26**	143.20 ± 5.40**	32.80 ± 1.39**
S. creatinine	0.45 ± 0.03**	1.85 ± 0.36	0.88 ± 0.04*	0.81 ± 0.03*	0.44 ± 0.03**
S. cholesterol	34.00 ± 1.74**	84.00 ± 4.94	99.20 ± 5.23	72.60 ± 3.43**	32.20 ± 2.49**
S. triglyceride	33.40 ± 3.45**	123.00 ± 6.63	93.20 ± 4.61*	94.60 ± 4.81*	38.20 ± 1.90**
HDL	24.60 ± 1.47**	10.20 ± 1.12	14.00 ± 0.65**	15.20 ± 2.44**	25.80 ± 0.98**
LDL	22.00 ± 2.10**	58.80 ± 3.22	42.20 ± 1.24**	34.20 ± 1.61**	23.60 ± 1.90**
Hemoglobin	11.20 ± 0.33**	6.90 ± 0.41	7.30 ± 0.45	8.80 ± 0.68*	10.90 ± 0.47**
Gly. hemoglobin	1.92 ± 0.17**	5.70 ± 0.37	4.20 ± 0.38*	3.20 ± 0.44**	2.00 ± 0.15**

Each value represents the mean ± S.E.M. of five observations.

* *P*<0.05,

** *P*<0.01 vs diabetic control (ANOVA followed by Dunnett's test)

### Collection of blood and estimation of biochemical parameters

The blood sugar level was measured using Accu-chek Active™ Test strips in Accu-chek Active™ Test meter by collecting the blood from rat tail vein. For other plasma profiles, blood was collected from retro-orbital plexus of the rats, under light ether anesthesia, using capillary tubes into eppendorf tubes containing heparin. The plasma was separated by centrifugation (5 min, 5000 rpm) and was analyzed for lipid profiles (serum cholesterol, serum triglyceride, HDL cholesterol, LDL cholesterol), serum creatinine, serum urea, hemoglobin and glylosylated hemoglobin. The plasma profiles were measured by standard enzymatic methods, with an automatic analyzer (Tulip, Goa, Model No. Evaluation 300).[14] and glycosylated hemoglobin by colorimetric method.

### Statistical analysis

The values are expressed as mean ± SEM. The results were analyzed for statistical significance using one-way ANOVA, followed by Dunnet's test. *P*<0.05 was considered significant.

## Results

### Preliminary phytochemical screening

The percentage yields of petroleum ether, chloroform, acetone, ethanol, aqueous and crude aqueous extracts were found to be 0.40, 6.40, 2.00, 4.10, 10.20 and 12.00% w/w respectively. The petroleum ether contained fats and terpenoids. The chloroform extract contained steroids and alkaloids. Acetone extract contained only tannins. Ethanol extract contained carbohydrates, flavonoids, alkaloids and tannins. Aqueous extract contained carbohydrates, alkaloids, flavonoids and saponins. Crude aqueous extract contained carbohydrates, alkaloids, flavonoids, tannins and saponins.

### The effect of extracts in glucose loaded hyperglycemic animals

Table 1 shows the antihyperglycemic effect in glucose loaded hyperglycemic rats, after administration of the plant extracts at a dose of 400 mg/kg. After 0.5 h of the glucose load, there was a significant rise in the blood glucose levels of the control animals, and at the end of two hours, the glucose level declined. Ethanolic extract exhibited significant antihyperglycemic activity at 1, 2 and 3 h after glucose load, compared to control. Crude aqueous extract and petroleum ether extract exhibited an effect only at 1 h. The remaining extracts did not show significant activity.

**Table 1:** Effect of various extracts of *M. champaca* in glucose loaded hyperglycemic rats

*Treatments*	*Dose mg/kg*	*Blood glucose concentration (mg/dl)*				
		
		*0 h*	*0.5 h*	*1 h*	*2 h*	*3 h*
Gluc. control	--	89.80 ± 3.02	144.40 ± 4.85	150.60 ± 4.01	124.40 ± 3.32	105.20 ± 4.77
Glibenclamide	5	90.20 ± 3.59	105.20 ± 3.49**	92.20 ± 4.60**	78.40 ± 4.20**	66.20 ± 3.68**
MC-P	400	90.80 ± 4.18	128.80 ± 5.74	125.40 ± 4.38*	108.20 ± 6.32	98.40 ± 3.22
MC-C	400	88.80 ± 4.28	144.40 ± 5.80	131.80 ± 4.22	122.20 ± 6.77	95.20 ± 4.84
MC-A	400	84.40 ± 4.41	141.20 ± 5.88	138.20 ± 5.20	120.80 ± 5.86	96.80 ± 4.83
MC-E	400	88.20 ± 3.18	111.80 ± 5.20*	109.20 ± 3.20**	85.40 ± 3.12**	85.60 ± 3.88*
MC-Aq	400	90.80 ± 4.88	166.20 ± 6.20	144.20 ± 5.20	124.60 ± 4.28	100.20 ± 3.84
MC -CAq	400	88.60 ± 4.24	134.20 ± 4.86	121.40 ± 5.22*	110.40 ± 4.88	90.20 ± 5.20

Each value represents the mean ± S.E.M. of five observations.

* *P*<0.05,

** *P*<0.01 vs control. MC - *Michelia champaca*, P - Petroleum ether (60-80o), C - Chloroform, A - Acetone, E - Ethanol, Aq - Aqueous, CAq - Crude aqueous

### The effect of ethanolic extract in fasted normal rats

Based on the antihyperglycemic activity, the active ethanolic extract was subjected to hypoglycemic studies at two dose levels (200 and 400 mg/kg) and the results are given in Table 2. The ethanolic extract of *M. champaca* did not show any hypoglycemic activity.

### The effect of ethanolic extract in alloxan induced diabetic rats

The basal blood glucose levels of all the groups were statistically not different from each other. Three days after alloxan administration, blood glucose values were five-folds higher in all the groups and were not statistically different from each other. After seven days, the values of blood glucose were decreased in all the treatment groups and the diabetic rats showed a slight increase in blood glucose level. The administration of plant extract and glibenclamide to diabetic rats restored the level of blood glucose significantly (*P*<0.01).

The level of total hemoglobin, glycosylated hemoglobin, serum urea, serum creatinine and lipid profiles of different experimental groups are represented in Table 3. The diabetic rats showed a significant decrease in the level of total hemoglobin and a significant increase in the level of glycosylated hemoglobin. The administration of plant extract and glibenclamide to diabetic rats restored the changes in the level of total hemoglobin and glycosylated hemoglobin to near normal levels (*P*<0.05) (*P*<0.01). But 200 mg/kg of ethanolic extract did not show significant effect in increasing the total hemoglobin content.

Alloxan induced diabetic rats showed significant hypercholesterolemia, as compared with control. Treatment with plant extract (400 mg/kg) showed significant decrease in cholesterol levels (*P*<0.01); at the same time, it showed an increase in HDL-c. Hypercholesterolemia was associated with hypertriglyceridemia, as compared with the control animals. Hypertriglyceridemia was also significantly prevented by treatment with the plant extract (*P*<0.05). Diabetic control rats showed a significant increase in creatinine and urea levels, as compared with the control animals. Treatment with ethanolic extract of *M. champaca* significantly decreased these values (*P*<0.01) (*P*<0.05). The extract was found effective in alleviating experimental diabetes and diabetes related complications [Table 3].

## Discussion

The present study was undertaken to examine the antidiabetic activity of *M. champaca* and to find out the active antihyperglycemic extract prepared using various solvents of this plant. Also, the antihyperglycemic activities of extracts obtained by successive solvent extraction method and crude aqueous extract prepared in a traditional manner were compared. The effect of this plant in diabetes changes in associated complications biochemical parameters was also assessed.

Effective blood glucose control is the key for preventing or reversing diabetic complications and improving the quality of life in patients with diabetes. Thus, sustained reduction in hyperglycemia will decrease the risk of developing microvascular complications and most likely reduce the risk of macrovascular complications.[20] On the basis of this statement, we have selected the glucose induced hyperglycemic model to screen the antihyperglycemic activity of the plant extracts.

In the glucose loaded hyperglycemic model, the plant tested for antihyperglycemic activity exhibited significant antihyperglycemic activity at a dose level of 400 mg/kg. Excessive amount of glucose in the blood induces the insulin secretion. This secreted insulin will stimulate peripheral glucose consumption and control the production of glucose through different mechanisms.[21] However, from the study (glucose control), it was clear that the secreted insulin requires 2-3 h to bring back the glucose level to normal. In case of the ethanolic extract, petroleum ether extract, crude aqueous extract and drug treated groups, the glucose levels did not exceed the control group, giving an indication regarding the supportive action of the extracts and drug in the glucose utilization. The most active antihyperglycemic ethanolic extract, when tested for hypoglycemic activity, did not exhibit hypoglycemic activity, suggesting its mechanism being similar to biguanides. Biguanides do not increase insulin secretion; they promote tissue glucose uptake and reduce hepatic glucose output, thereby producing antihyperglycemic effect and not hypoglycemic effect.[21]

Alloxan produces hyperglycemia by selective cytotoxic effect on pancreatic beta cells. Our investigations indicate the efficiency of the plant extract in the maintenance of blood glucose levels in alloxan induced diabetic rats, may be possibly by the above mentioned mechanism. In uncontrolled or poorly controlled diabetes, the excess glucose present in the blood reacts with hemoglobin. Therefore, the total hemoglobin level is decreased and glycosylated hemoglobin is increased in alloxan diabetic rats.[22] Administration of ethanolic extract for seven days prevented a significant elevation in glycosylated hemoglobin, thereby increasing the level of total hemoglobin (*P*<0.05) (*P*<0.01) in diabetic rats. This could be due to the result of improved glycemic control produced by the plant extract.

The levels of serum lipids are usually elevated in diabetes mellitus and such an elevation represents a risk factor for coronary heart disease. This abnormally high level of serum lipids is mainly due to the uninhibited actions of lipolytic hormones on the fat depots, mainly due to the action of insulin. Under normal circumstances, insulin activates the enzyme lipoprotein lipase, which hydrolyses triglycerides. However, in diabetic state, lipoprotein lipase is not activated due to insulin deficiency, resulting in hypertriglyceridemia[23] and insulin deficiency is also associated with hypercholesterolemia due to metabolic abnormalities.[24] In our study also, the diabetic rats showed hypercholesterolemia and hypertriglyceridemia and the treatment with plant extract significantly decreased both cholesterol and triglyceride levels. This implies that the ethanolic extract of *M. champaca* flower buds may prevent or be helpful in reducing the complications of lipid profile seen in some diabetics in whom hyperglycemia and hypercholesterolemia coexist quite often.[25]

The diabetic hyperglycemia induced by alloxan produces elevation of plasma levels of urea and creatinine, which are considered significant markers of renal dysfunction.[26] After treatment of alloxan-diabetic rats with ethanolic extract, the level of urea and creatinine were significantly decreased, compared to the mean value of diabetic group. This further suggests the potential utility of these plants in diabetes associated complications.[27]

Mostly, it was believed that the formation of the artefacts during the preparation of crude aqueous extracts would be responsible for the biological activities.[28] In our study, the crude aqueous extract of *M. champaca* was found active only at 1 h after glucose administration. Though the main classes of active constituents are present in the crude aqueous extract of this plant, no significant activity was observed. This may be due to the lower concentrations of active constituents present at 400 mg/kg of crude aqueous extracts, when compared to 400 mg/kg of successively fractionated extracts. Babayi *et al.*[29] suggested the same for the better activity of fractions, compared to extracts. However, the reason behind the less activity of crude aqueous extract was unknown. Hence, the antidiabetic activity of the extracts was caused by substances that naturally exist in the plant, and not due to transformations induced by heating.

We conclude that the plant has shown potential activity in decreasing the serum glucose level and other complications associated with experimental diabetes. This research supports the inclusion of this plant in traditional antidiabetic preparations.
